# Associations of comorbid substance use disorders with clinical outcomes in schizophrenia using electronic health record data

**DOI:** 10.1016/j.schres.2023.08.023

**Published:** 2023-10

**Authors:** Rashmi Patel, Kelly M.Y. Chan, Emily O.C. Palmer, Matthew Valko, Guruprabha Guruswamy, Sheryl Ker, Gunjan Batra, Miguel E. Rentería, Scott H. Kollins

**Affiliations:** a King's College London, Institute of Psychiatry, Psychology and Neuroscience, London, UK; b Holmusk Technologies Inc., New York, NY, USA

**Keywords:** Real-world evidence, Illness severity, Treatment patterns, Treatment discontinuation, Hospital utilization, NeuroBlu

## Abstract

**Background and hypothesis:**

Schizophrenia and comorbid substance use disorders (SUDs) are associated with poor treatment outcomes but differences between the associations of different SUDs with clinical outcomes are poorly characterized. This study examines the associations of comorbid SUDs with clinical outcomes in schizophrenia using a largescale electronic health record (EHR) database.

**Design:**

Real-world data (RWD) analysis using the NeuroBlu database; de-identified EHR data were analysed. Multivariable logistic regression, Poisson and CoxPH models were used to compare the associations of specific comorbid SUDs with outcome variables.

**Results:**

Comorbid SUD was significantly different on all outcome measures compared to no SUD (*U* = 1.44e^7^–1.81e^7^, all *p*s < .001), except number of unique antipsychotics (*U* = 1.61e^7^, *p* = .43). Cannabis (OR = 1.58, *p* < .001) and polysubstance (OR = 1.22, *p* = .007) use disorders were associated with greater CGI-S. Cannabis (IRR = 1.13, *p* = .003) and polysubstance (IRR = 1.08, *p* = .003) use disorders were associated with greater number of unique antipsychotics prescribed, while cocaine (HR = 1.87, *p* < .001), stimulants (HR = 1.64, *p* = .024), and polysubstance (HR = 1.46, *p* < .001) use disorders were associated with a shorter time to antipsychotic discontinuation. Conversely, alcohol use (IRR = 0.83, *p* < .001), cocaine use (IRR = 0.61, *p* < .001), opioid use (IRR = 0.61, *p* < .001), stimulant use (IRR = 0.57, *p* < .001) and polysubstance use (IRR = 0.87, *p* < .001) disorders were associated fewer inpatient days.

**Conclusion:**

Comorbid SUDs were generally associated with greater CGI-S and poorer clinical outcomes in patients with schizophrenia. Treatment strategies should target not only schizophrenia symptoms but also comorbid SUD to improve management of both conditions.

## 1 Introduction

Schizophrenia is a chronic and severe mental illness with a global prevalence of 1 % and defined by the National Institute of Mental Health as a “mental disorder characterized by impairments in thought processes, perceptions, emotional responsiveness, and social interactions.” (National Institute of Mental Health, 2022; Moreno-Küstner et al., 2018) Patients with schizophrenia experience disabling symptoms and reduced functioning, resulting in treatment costs that are a major economic burden on US healthcare systems and estimated at $155.70 billion per year (Cloutier et al., 2016). This is, in part, due to high rates of psychiatric comorbidity associated with schizophrenia. It has been estimated that 50 % of patients who received a diagnosis of schizophrenia suffer from at least one additional comorbid psychiatric disorder (Pasic et al., 2005). The most prevalent comorbidity is substance use disorder (SUD), impacting 42–49 % of patients. The most common SUDs include nicotine (60–95 %), alcohol (20–86 %), cannabis (12–80 %), and cocaine (15–50 %) (Pasic et al., 2005). In addition, polysubstance use disorder has been identified as a common feature in patients, where alcohol, cannabis, and other substances were likely to be used concurrently with each other (Brady and Sinha, 2005; Kerner, 2015; Moore et al., 2012).

Schizophrenia with comorbid SUD is associated with higher rates of relapse and rehospitalization compared to schizophrenia without SUD (Kessler and Lev-Ran, 2019). In older adults, comorbid SUDs were associated with longer length-of-stay in hospital and non-psychiatric medical comorbidities (Lane et al., 2018; Wu et al., 2015). A recent meta-analysis indicated that schizophrenia with comorbid cannabis use disorder was associated with less adherence to medication compared with non-cannabis users (Foglia et al., 2017). Despite poor treatment outcomes associated with schizophrenia with comorbid SUDs, there is a lack of research on the effectiveness of pharmacological treatments and factors supporting treatment adherence for this specific patient group. In fact, patients with schizophrenia and comorbid SUDs are often excluded from pharmacological trials to avoid confounding effects and high attrition rates (Wobrock and Soyka, 2008).

This study aimed to examine the prevalence and associated clinical outcomes of schizophrenia with comorbid SUDs using real-world data (RWD). RWD are collected from a variety of sources, such as electronic health records (EHRs), reflecting patients' health status and relevant information routinely collected in healthcare settings (Patel et al., 2022). As such, real-world evidence (RWE) generated from RWD could provide valuable information about clinical symptoms and outcomes observed in real-world practice, and may complement other sources of evidence such as clinical trials (Busner and Targum, 2007). Additionally, we aimed to examine which substance-specific SUDs were associated with poorer clinical outcomes. We hypothesized that schizophrenia with comorbid SUDs would be associated with poorer clinical outcomes — namely, higher scores on Clinical Global Impression-Severity (CGI-S), a greater number of days spent in a psychiatric hospital, shorter time to the first discontinuation of antipsychotics, and more unique antipsychotics prescribed compared with schizophrenia without comorbid SUDs.

## 2 Methods

### 2.1 Source of clinical data

This study leveraged de-identified electronic health record (EHR) data from the NeuroBlu Research Database (NeuroDB), a longitudinal mental and behavioral health dataset comprising both structured and unstructured clinical data at individual patient level (Patel et al., 2022). At the time of this study, NeuroDB included data from 25 mental and behavioral health centers across the US spanning over 20 years, including both inpatient and outpatient settings. All data were de-identified using the Safe Harbor standards outlined in the HIPAA Privacy Law. Institutional Review Board (IRB) evaluation with a waiver of informed consent was obtained prior to study commencement. As EHRs contain observational, routinely-collected data, a RECORD checklist was completed as part of the reporting of this study (Appendix A).

### 2.2 Participants

The cohort included patients aged 18 years or older with a diagnosis of schizophrenia (ICD-10 F20*) before January 2019. Patients were excluded from the cohort if their records provided insufficient follow-up data or had no recorded gender. All included patients had at least 12 months of follow-up data available (defined as at least one clinical encounter 12 months following the index date of first recorded schizophrenia diagnosis). A total of 13,634 individuals were included in the cohort (see Supplementary Fig. 1 for details of cohort attrition). Of these, 3010 (22.1 %) patients were identified as having a comorbid SUD where there was a record of a SUD diagnosis (ICD9/10 codes listed in Supplementary Table 7) before or at the same time as their diagnosis of schizophrenia. These patients were further divided based on specific SUD diagnoses (Table 1). Since the prevalence rates of hallucinogen and inhalant use disorders were low, they were combined with other less common psychoactive SUDs to form the category “others” in subsequent analyses. Additionally, several patients had more than one SUD diagnosis and were accounted for in multiple SUD categories (Supplementary Fig. 2).

**Table 1:** Characteristics of patients with schizophrenia diagnosis (n = 13,634) with and without comorbid substance use disorder (SUD).^a^

Characteristics	Patients with schizophrenia			
	Total (n = 13,634)	With SUD (n = 3010)	Without SUD (n = 10,624)	With vs. without SUD
	*M* (*SD*)	*M* (*SD*)	*M* (*SD*)	*U* (*p*)
Age (years)	41.5 (15.0)	36.8 (12.9)	42.9 (15.2)	1.24e^7^ (<.001)

	n (%)	n (%)	n (%)	χ^2^ (*p*)
Race				42.00 (<.001)
White	5280 (38.7)	1085 (36.1)	4195 (39.5)	
Black or African American	4259 (31.2)	1036 (34.4)	3223 (30.3)	
Native Hawaiian or Pacific Islander	245 (1.8)	52 (1.7)	193 (1.8)	
Asian	207 (1.5)	21 (0.7)	186 (1.8)	
American Indian or Alaska Native	46 (0.3)	10 (0.3)	36 (0.3)	
Others	504 (3.7)	94 (3.1)	410 (3.9)	
Not known	3093 (22.7)	712 (23.7)	2381 (22.4)	
Gender				333.00 (<.001)
Male	8571 (62.9)	2320 (77.1)	6251 (58.8)	
Female	5063 (37.1)	690 (22.9)	4373 (41.2)	
Marital status				28.98 (<.001)
Single	8694 (63.8)	1948 (64.7)	6746 (63.5)	
Married/Engaged/In Relationship	1066 (7.8)	199 (6.6)	867 (8.2)	
Divorced	807 (5.9)	200 (6.6)	607 (5.7)	
Separated	345 (2.5)	94 (3.1)	251 (2.4)	
Widowed	237 (1.7)	30 (1.0)	207 (2.0)	
Not known	2485 (18.2)	539 (17.9)	1946 (18.32)	
Employment status				74.00 (<.001)
Employed	579 (4.3)	132 (4.4)	447 (4.2)	
Disabled	1100 (8.1)	232 (7.7)	868 (8.2)	
Unemployed	2135 (15.7)	609 (20.2)	1526 (14.4)	
Student	130 (1.0)	31 (1.0)	99 (0.9)	
Retired	112 (0.8)	9 (0.3)	103 (1.0)	
Not known	9578 (70.3)	1997 (66.4)	7581 (71.4)	
Year of schizophrenia diagnosis				26.00 (<.001)
Before 2005	586 (4.3)	179 (6.0)	407 (3.8)	
2005 to 2009	3375 (24.8)	724 (24.1)	2651 (25.0)	
2010 to 2014	6941 (50.9)	1526 (50.7)	5415 (51.0)	
2015 to 2019	2732 (20.0)	581 (19.3)	2151 (20.3)	
Year of SUD diagnosis				1.36e^4^ (<.001)
Before 2005	–	235 (7.8)	–	
2005 to 2009	–	795 (26.4)	–	
2010 to 2014	–	1546 (51.4)	–	
2015 to 2019	–	434 (14.4)	–	
Types of SUD^b^				
Alcohol	–	1204 (40.0)	–	
Cannabis	–	988 (32.8)	–	
Cocaine	–	506 (16.8)	–	
Opioid	–	280 (9.3)	–	
Nicotine	–	252 (8.3)	–	
Stimulant	–	158 (5.2)	–	
Sedative, hypnotic or anxiolytic	–	62 (2.1)	–	
Hallucinogen	–	43 (1.4)	–	
Inhalant	–	1 (0.03)	–	
Others	–	1129 (37.5)	–	

Abbreviations: SD, standard deviation; SUD, substance use disorder.

a Chi-square tests (for categorical variables) and Mann Whitney U tests (for continuous variables) were used to compare characteristics between patients with and without comorbid SUDs; *level of significance*, *α* = 0.05.

b The total count of this section is greater than the number of patients identified with schizophrenia and a comorbid SUD as patients may be diagnosed with >1 SUD and thus accounted for in multiple categories.

### 2.3 Clinical outcome measures and covariates

The primary outcome measure was illness severity determined by CGI-S scale scores documented within 30 days of the first-recorded schizophrenia diagnosis (index date). The CGI-S scale is frequently utilized in clinical practice to quantify and track patient progress over time. Using a seven-point scale, clinicians record their view of a patient's illness severity where a score of 1 represents “normal”, and 7 represents “among the most extremely ill patients” (Busner and Targum, 2007). Secondary outcome measures were observed for a duration of one year from the index date (follow-up period), and included the number of inpatient days, time taken until the first discontinuation of antipsychotics, and number of unique antipsychotic medications prescribed. These were identified as important indicators of illness severity and treatment discontinuation because they could be seen as proxy measures of effectiveness, and tolerability of psychotropic treatment, as well as rates of treatment failure (Patel et al., 2016).

Covariates considered included age, gender, race, marital status, employment status, and year of first schizophrenia diagnosis. These demographic factors were chosen as they were previously associated with the risk of patients developing a comorbid SUD (Dixon, 1999). Year of diagnosis was included because it may have influenced prescribing trends and availability of certain antipsychotic medications. The main analyses were also adjusted for the clinic centers at which patients received care.

### 2.4 Statistical analysis

Two primary comparisons were of interest to delineate the effects of comorbid SUDs: 1) those with a comorbid SUD versus without SUD, and 2) those with presence of a specific comorbid SUD versus absence of the same SUD. Results of the first comparison are presented in this manuscript, while results from the second comparison are provided in the supplementary materials (Supplementary Tables 3–6).

Chi-square tests (categorical variables) and Mann Whitney *U* tests (continuous variables) were used to compare outcome measures between patients with and without comorbid SUDs. Further analyses were conducted to provide greater clarity of the effects of specific comorbid SUDs on illness severity and treatment discontinuation. Baseline CGI-S scale scores (“1–4” and “5–7”) were dichotomized into two groups based on the mean CGI-S score of 4.3, where the latter group (“5–7”) indicated greater illness severity. Multivariable logistic regression analyses were then conducted to explore associations between specific comorbid SUDs with CGI-S scores. A Poisson distribution model was used to assess the differences in number of inpatient days and number of unique antipsychotics prescribed between specific SUDs. Comparisons between SUDs and the time taken to first antipsychotic discontinuation were explored using a Cox proportional hazard (CoxPH) model (Cox, 1972).

### 2.5 Missing data

Data from certain variables were missing due to lack of clinical documentation in the EHR dataset. Patients were excluded (n = 26) if their gender was not known; while patients with unknown or missing data for other demographic variables, such as race, marital status, and employment status, were classified as a separate category labelled as “not known”. In some cases, demographic groups were consolidated due to small sample sizes to provide meaningful information to the models. This was done in the case of age (grouped into <40 years and ≥40 years based on the bimodal distribution of the data), race, marital status, and employment status.

## 3 Results

### 3.1 Descriptive statistics

The total cohort of this study comprised 13,634 patients (male: n = 8571, 62.9 %). 3010 (22.1 %) had a least one comorbid SUD recorded (male: n = 2320, 77.1 %). The most prevalent racial groups represented were White (n = 5280, 38.7 %) and Black or African American (n = 4259, 31.2 %), while 22.7 % of the cohort did not have known information regarding their race. The majority of the cohort was single (n = 8694, 63.8 %) and without a known employment status (n = 9578, 70.3 %). Most patients were first diagnosed with schizophrenia between 2010 and 2014 (n = 6941, 50.9 %), and the most prevalent comorbid SUD of the total cohort was alcohol (n = 1204, 40 %), followed by cannabis (n = 988, 32.8 %), and cocaine (n = 506, 16.8 %). Patients with comorbid SUDs were also likely to be younger (*M* *=* 36.8 years, *SD* = 12.9) than those without a comorbid SUD (*M* = 42.9 years, *SD* = 15.2) (Table 1).

### 3.2 Comparing schizophrenia with or without comorbid SUD on clinical outcomes

Of the total cohort, 12,676 (93.0 %) had CGI-S data recorded at baseline. Those patients with a comorbid SUD had greater CGI-S scores (*M* = 4.4, *SD* = 1.7, *Median* = 5.0, *IQR* = 2.0) than those without (*M* = 4.3, *SD* = 1.7, *Median* = 5.0, *IQR* = 1.0, *p* < .001). Patients with comorbid SUDs also had a shorter time to first discontinuation of antipsychotics (*M* = 176.8 days, *SD* = 393.3; without SUD: *M* = 270.0 days, *SD* = 538.1, *p* < .001), and fewer inpatient days (*M* = 7.5, *SD* = 15.5; without SUD: *M* = 7.6, *SD* = 20.5, *p* < .001). There was no significant difference in the number of antipsychotics prescribed (SUD: *M* = 1.4, *SD* = 1.2; without SUD: *M* = 1.4, *SD* = 1.1) (Table 2).

**Table 2:** Outcome variables for patients with a schizophrenia diagnosis with or without comorbid substance use disorder (SUD).^a^

Outcome variables	Patients with a schizophrenia diagnosis			
	Total	with SUD	without SUD	with vs. without SUD
	*M* (*SD*)	*M* (*SD*)	*M* (*SD*)	*U* (*p*)
No. of inpatient days	7.6 (19.5)	7.5 (15.5)	7.6 (20.5)	1.81e^7^ (<.001)
No. of unique antipsychotics prescribed	1.4 (1.2)	1.4 (1.2)	1.4 (1.1)	1.61e^7^ (=.43)
Time to first antipsychotic discontinuation (days)	249.4 (511.1)	176.8 (393.3)	270.0 (538.1)	1.44e^7^ (<.001)
CGI-S score (continuous)	4.3 (1.7)	4.4 (1.7)	4.3 (1.7)	1.70e^7^ (<.001)

	*Median* (*IQR*)	*Median* (*IQR*)	*Median* (*IQR*)	
	5.0 (1.0)	5.0 (2.0)	5.0 (1.0)	

	n (%)	n (%)	n (%)	χ^2^ (*p*)
CGI-S score (categorical)				49.50 (<.001)
1	364 (2.7)	85 (2.8)	279 (2.6)	
2	326 (2.4)	69 (2.3)	257 (2.4)	
3	1175 (8.6)	194 (6.5)	981 (9.2)	
4	3743 (27.5)	750 (24.9)	2993 (28.2)	
5	3828 (28.1)	885 (29.4)	2943 (27.7)	
6	2985 (21.9)	744 (24.7)	2241 (21.1)	
7	255 (1.9)	66 (2.2)	189 (1.8)	
Missing/unavailable	958 (7.0)	217 (7.2)	741 (7.0)	

Abbreviations: CGI-S, Clinical Global Impression-Severity; SD, standard deviation; IQR, interquartile range; M, mean; U, Mann-Whitney *U* test statistic; No., number.

a Chi-square tests (for categorical variables) and Mann Whitney U tests (for continuous variables) were used to compare characteristics between patients with and without comorbid SUDs; *level of significance*, *α* = 0.05.

### 3.3 Comparing effects of specific comorbid SUDs on clinical outcomes

#### 3.3.1 Outcome variable: baseline CGI-S scores (“1–4” and “5–7”)

Further analyses were conducted to explore the associations between substance-specific comorbid SUDs and clinical outcomes. Multivariable logistic regression analyses corroborated the initial finding that the presence of a comorbid SUD was associated with higher baseline CGI-S scores (“5–7”) indicating greater illness severity. Specifically, comorbid use of cannabis (OR = 1.58, 95%CI [1.23, 2.04], *p* < .001), polysubstance (OR = 1.22, 95%CI [1.06, 1.42], *p* = .007) and other less common SUDs (OR = 1.41, 95%CI [1.18, 1.70], *p* < .001) were associated with higher CGI-S scores at baseline compared with the absence of comorbid SUDs (Table 3).

**Table 3:** Logistic regression comparing patients with and without comorbid substance use disorders (SUD) on baseline CGI-S scores^a^ (n = 12,676).

Variables	n	Mean (*SD*)	Odds ratio	Confidence interval	*p*	Reference category	
						n	Mean (*SD*)
SUD category						*Without SUD*	
Alcohol only	492	4.4 (1.5)	1.03	0.84 to 1.26	.756	9883	4.6 (1.2)
Cannabis only	336	4.9 (1.1)	1.58	1.23 to 2.04	<.001		
Opioid only	111	4.2 (1.2)	0.67	0.43 to 1.03	.065		
Cocaine only	104	4.6 (1.2)	1.17	0.77 to 1.79	.456		
Nicotine only	55	4.6 (1.4)	1.22	0.68 to 2.19	.503		
Stimulant only	42	5.0 (0.9)	1.97	0.92 to 4.21	.079		
Others only	642	5.0 (1.1)	1.41	1.18 to 1.70	<.001		
Polysubstance	1011	4.7 (1.2)	1.22	1.06 to 1.42	.007		
Gender						*Female*	
Male	7939	4.6 (1.2)	0.96	0.89 to 1.04	.348	4737	4.6 (1.2)
Age^b^						*Age < 40*	
Age 40 and above	6725	4.5 (1.3)	0.79	0.73 to 0.86	<.001	5951	4.7 (1.2)
Race						*Black*	
White	4890	4.6 (1.2)	1.18	1.07 to 1.31	.002	3848	4.6 (1.2)
Others	988	5.1 (1.1)	1.31	1.10 to 1.55	.003		
Unknown	2950	4.4 (1.3)	1.34	1.19 to 1.5	<.001		
Marital status						*Divorced*/*separated*	
Single	8123	4.7 (1.2)	0.99	0.85 to 1.14	.842	1068	4.4 (1.3)
Married/engaged/in a relationship	981	4.4 (1.2)	1.02	0.84 to 1.23	.865		
Widowed	208	4.4 (1.2)	1.24	0.91 to 1.70	.174		
Unknown	2296	4.4 (1.3)	0.95	0.8 to 1.12	.524		
Employment status						*Disabled*	
Employed	534	4.8 (1.2)	0.81	0.64 to 1.03	.091	1000	4.8 (1.1)
Unemployed/student/retired	2207	4.9 (1.1)	0.95	0.79 to 1.13	.541		
Unknown	8935	4.5 (1.3)	0.74	0.63 to 0.86	<.001		
Year of schizophrenia diagnosis						*2004 and before*	
2005–2009	3290	4.8 (1.2)	1.52	1.11 to 2.09	.008	216	4.8 (0.9)
2010–2014	6560	4.5 (1.3)	1.40	1.02 to 1.91	.037		
2015–2020	2610	4.5 (1.2)	1.15	0.83 to 1.60	.395		

a Baseline CGI-S scores were dichotomized to represent low scores (1–4) and high scores (5–7); level of significance, α = 0.05.

b In a sensitivity analysis, age was modelled in a generalized additive model to model age non-linearly (Supplementary Fig. 3). Note: The analysis was also adjusted for clinic ID.

#### 3.3.2 Outcome variable: number of inpatient days

A Poisson distribution model revealed that comorbid alcohol use (IRR = 0.83 95 % CI [0.80, 0.86], *p* < .001), cocaine use (IRR = 0.61, 95 % CI [0.56, 0.67], *p* < .001), opioid use (IRR = 0.61, 95 % CI [0.55, 0.68], *p* < .001), stimulant use (IRR = 0.57, 95 % CI [0.51, 0.63], *p* < .001) and polysubstance use (IRR = 0.87, 95 % CI [0.85, 0.89], *p* < .001) were associated with fewer inpatient days compared with the absence of comorbid SUD (Table 4). Other less common SUDs (IRR = 1.03, 95%CI [1.00. 1.05], *p* = .034) were associated with more inpatient days, though with a small effect size.

**Table 4:** Poisson distribution model comparing the impact of comorbid substance use disorders (SUD) on the number of inpatient days of patients with schizophrenia (n = 13,634), a significance threshold of *p* < .05 was used.

Variables	n	Mean (SD)	IRR	Confidence interval	*p*	Reference category	
						n	Mean (SD)
SUD category						*Without SUD*	
Alcohol only	540	5.7 (12.8)	0.83	0.80 to 0.86	<.001	10,624	7.6 (20.5)
Cannabis only	363	8.6 (18.0)	0.99	0.95 to 1.03	.585		
Cocaine only	135	3.7 (8.1)	0.61	0.56 to 0.67	<.001		
Opioid only	113	2.7 (5.9)	0.61	0.55 to 0.68	<.001		
Nicotine only	59	8.1 (17.0)	1.01	0.93 to 1.11	.78		
Stimulant only	42	7.5 (8.4)	0.57	0.51 to 0.63	<.001		
Others only	667	10.7 (18.9)	1.03	1.00 to 1.05	.034		
Polysubstance	1091	7.1 (14.6)	0.87	0.85 to 0.89	<.001		
Gender						*Female*	
Male	8571	7.6 (19.8)	0.96	0.95 to 0.97	<.001	5063	7.5 (19.1)
Age^a^						*Age < 40*	
Age 40 and above	7252	7.2 (20.1)	1.01	1.00 to 1.03	.074	6382	8.0 (18.9)
Race						*Black*	
White	5280	7.2 (19.3)	1.19	1.17 to 1.21	<.001	4259	7.8 (18.9)
Others	1002	16.4 (29.7)	1.19	1.16 to 1.21	<.001		
Unknown	3093	5.1 (15.4)	1.00	0.98 to 1.03	.78		
Marital status						*Divorced*/*separated*	
Single	8694	9.8 (22.5)	1.15	1.11 to 1.19	<.001	1152	3.6 (9.4)
Married/engaged/in a relationship	1066	3.3 (9.6)	1.05	1.01 to 1.10	.03		
Widowed	237	4.6 (10.4)	1.12	1.05 to 1.20	<.001		
Unknown	2485	3.9 (13.9)	0.95	0.91 to 0.99	.01		
Employment status						*Disabled*	
Employed	579	6.5 (12.5)	0.62	0.59 to 0.64	<.001	1100	9.6 (21.5)
Unemployed/student/retired	2377	13.8 (26.7)	1.04	1.01 to 1.06	.002		
Unknown	9578	5.9 (17.0)	0.70	0.69 to 0.72	<.001		
Year of Schizophrenia diagnosis						*2004 and before*	
2005–2009	3375	8.3 (21.5)	1.18	1.12 to 1.24	<.001	586	4.1 (8.3)
2010–2014	6941	7.3 (19.8)	1.64	1.56 to 1.72	<.001		
2015–2020	2732	8.3 (17.8)	1.42	1.35 to 1.49	<.001		

a In a sensitivity analysis, age was modelled in a generalized additive model to model age non-linearly (Supplementary Fig. 4). Note: IRR: Incidence rate ratio. The analysis was also adjusted for clinic ID.

#### 3.3.3 Outcome variable: unique number of prescribed antipsychotics

A Poisson distribution model found that comorbid cannabis use (IRR = 1.13, 95%CI [1.04, 1.23], *p* = .003) or polysubstance use disorder (IRR = 1.08, 95%CI [1.03, 1.14], *p* = .003) were associated with an increased number of unique prescribed antipsychotics (Supplementary Table 1). This finding suggests that patients with schizophrenia and these comorbid SUDs may have higher treatment failure compared with those without SUDs.

#### 3.3.4 Outcome variable: time to first antipsychotic discontinuation

A CoxPH model found that comorbid cocaine (HR = 1.87, 95%CI [1.42, 2.45], *p* < .001), stimulant (HR = 1.64, 95%CI [1.07, 2.52], *p* = .024), and polysubstance (HR = 1.46, [1.33, 1.59], *p* < .001) use disorders were associated with shorter time to first antipsychotic discontinuation than those without comorbid SUD (Supplementary Table 2).

## 4 Discussion

Analysis of a large-scale EHR dataset demonstrated that 22.1 % of patients with schizophrenia had a comorbid SUD diagnosis and that this comorbidity was associated with poorer clinical outcomes when compared with an absence of comorbid SUD. Though this prevalence is in line with previous findings (Fowler et al., 1998; Hunt et al., 2018; Pasic et al., 2005), SUDs are often under-reported and these prevalence rates could be an underestimation (Bahorik et al., 2014). Given the real-world nature of our data, comorbid SUD may also be underdiagnosed in clinical settings among patients with schizophrenia. Underdiagnosis could have crucial implications in the treatment of this sub-population, often associated with severe symptoms and high rates of suicidality (Buckley, 2006; Lambert et al., 2005; Turkington et al., 2009).

Our analyses comparing patients with schizophrenia and SUDs with those without SUDs yielded findings that were aligned with previous studies. Comorbid SUDs are often linked to poorer clinical outcomes, such as higher rates of relapse and rehospitalization (Kessler and Lev-Ran, 2019), which was also reflected by greater illness severity in our sample based on CGI-S scores. Our results also suggested the possibility of poorer treatment adherence for the group with comorbid SUDs based on higher number of unique antipsychotics prescribed and shorter times to treatment discontinuation compared to the group without SUDs. Our finding is consistent with a prospective cohort study of 135 patients with schizophrenia, which found that individuals with comorbid SUDs were eight times more likely not to not be adherent with recommended medications than those without SUDs (Owen et al., 1996). Other studies have found that schizophrenia with SUDs is not only related to poorer medication adherence but also poorer tolerance and response to medications on measures of total symptoms, positive and negative symptoms, general psychopathology, depression, and overall functioning (Green et al., 2004). This is likely to result in clinicians prescribing more unique antipsychotics in a bid to find the best combination for these patients.

On the other hand, schizophrenia with certain comorbid SUDs was found to be associated with fewer recorded inpatient days compared to without SUDs. While comorbid SUDs are often related to a higher risk of hospitalization, (Lähteenvuo et al., 2021) our data reflected the duration of psychiatric hospitalization specifically. A longitudinal 15-year follow-up study of 216 patients found that while frequency of hospitalization was higher for patients with a comorbid SUD, the length of psychiatric hospitalizations was shorter for the same group compared with those without a comorbid SUD (12 vs 21 days), (Schmidt et al., 2011) which aligned with our findings.

Our study also adds uniquely to the literature in exploring substance-specific comorbid SUD and its association to various indicators of illness severity and treatment effectiveness using RWE generated from EHR. In our sample, the three most prevalent SUDs were alcohol, cannabis, and cocaine. Of these, cannabis use disorder was associated with greater baseline illness severity and more unique antipsychotics prescribed, indicating poorer medication tolerance compared with schizophrenia without SUDs. This is in line with previous studies using EHR data which found that cannabis use disorder was a significant risk factor for medication non-adherence (Patel et al., 2016; Patel et al., 2020).

Few studies have examined schizophrenia with cocaine use disorder specifically; our finding that cocaine use disorder was associated with a shorter time to antipsychotic discontinuation is novel and aligned with dilemmas raised about treatment in this patient group (Beresford et al., 2005). Antipsychotics (traditionally dopamine blocking agents) are likely to cause a dearth of dopamine activity, particularly in cocaine users who often have disrupted receptor function as a result of chronic use of the dopaminergic drug (Zimmet et al., 2000). It is therefore unsurprising that this group of patients would discontinue antipsychotics at a greater rate, highlighting the need for interventions targeting both schizophrenia and cocaine use.

Among the more common forms of SUDs, we found that alcohol, cocaine, stimulants, and polysubstance use were associated with fewer inpatient days than patients without comorbid SUDs. This was aligned with our findings of comorbid SUD in general being associated with fewer inpatient days compared to the absence of comorbid SUDs, which has also been observed in previous literature. In a national register study Schmidt et al. found that patients with schizophrenia and co-occurring substance use disorder had a median duration of hospital admission of 12 days (IQR: 4–43) while patients with a schizophrenia diagnosis only had a median duration of hospital admission of 21 days (IQR6–70) (Schmidt et al., 2011). Ries et al. found that psychiatric hospital admission was 30 % shorter for patients with schizophrenia or schizoaffective disorder and co-occurring substance use disorder than patients without a co-occurring substance use disorder (Ries et al., 2000). It has been hypothesized that patients with schizophrenia and SUDs may stabilize more quickly during acute hospitalization compared to those without SUDs, as the substance abuse may temporarily amplify symptoms or that patients with comorbid SUDs are more likely to have schizophrenia with better prognosis (Ries et al., 2000). We further speculate that patients with comorbid SUDs are also more likely to be known to healthcare services, where the patient is already assessed and assigned to appropriate specialists, and a dual-focus treatment plan may have already been implemented resulting in more targeted care and faster recovery upon hospitalization.

### 4.1 Clinical implications

Using RWD, our study shows that patients with schizophrenia and comorbid SUDs have greater illness severity and poorer treatment effectiveness versus those without SUDs. While our analyses revealed statistically significant differences between subgroups of patients, some of these differences were small and may not be clinically meaningful. The association between schizophrenia and comorbid SUD has been frequently discussed in the literature given the high prevalence of comorbidity. One hypothesis posits that due to the impairment of dopaminergic activity in the central striatum in patients with schizophrenia, they may have a hypersensitive dopamine system that may contribute to their vulnerability toward substance use (Thompson et al., 2013). This may result in amplified responses to drug use, as previous studies have shown greater activation of a region within the brain reward circuit (i.e., ventromedial prefrontal cortex) in response to nicotine cues for smokers with schizophrenia compared with smokers without (Potvin et al., 2016). In addition, hypoconnectivity in the brain reward circuitry proposed as a common dysfunction in schizophrenia has also been found to be ameliorated by using cannabis which may account for the high frequency of overuse of these substances (Fischer et al., 2014).

Given the high prevalence of schizophrenia with comorbid SUDs, it is essential that treatment for this subgroup with dual diagnoses target symptoms of both disorders. Traditionally, interventions specific to mental health are different to that of SUD programs. Additionally, these interventions are not often provided within a single behavioral healthcare provider (De Witte et al., 2014), resulting in patients having to coordinate care between service providers leading to high rates of attrition. Our results highlight the importance for services to provide holistic care for patients, rather than specialized treatments targeting a single disorder, since comorbidities in mental health are often the norm rather than exception (Kessler et al., 2011).

Recent clinical guidelines (Campbell et al., 2023) in the United States have recommended the use of antipsychotic medication, instead of other primary medications, to treat schizophrenia symptoms with SUD-induced exacerbations. In instances where medication adherence is poor, long-acting injectable antipsychotic medications are preferred over daily oral-administered prescriptions. Additionally, a multimodal, integrated care for patients with schizophrenia and SUD is recommended over separate care for each disorder, which includes pharmacotherapy and one or more psychosocial interventions. The integrated care model involves a single clinician or team that treats both conditions; in cases where this is not available, it is recommended that clinicians treating the co-occurring conditions should coordinate care closely.

### 4.2 Strengths and limitations

There are multiple strengths of the present research. Firstly, of our cohort, 93.0 % had complete data on CGI-S scores, suggesting that the RWD examined in our analysis were valuable and robust. Second, the large cohort size suggests a representative sample of patients with schizophrenia receiving mental health care in the US, which allowed for the examination of important outcomes related to illness severity and treatment adherence in this patient group. Further, analyzing large RWD datasets suggests that even small effect sizes could have important implications for overall healthcare delivery when aggregated to the population. The richness of the data also allowed for the granular evaluation of substance-specific SUDs, which provided critical insight into the patient's groups that would most benefit from treatment targeted at both schizophrenia and SUD. The use of RWD also reflects generalizability of the results as it was derived from real-world settings rather than controlled clinical trials.

It was not possible to determine the contribution of other factors that could have significantly impacted treatment outcomes. For example, the dataset did not contain a full record of an individual patient's healthcare journey, and therefore the data did not reflect interactions that patients may have had across multiple services. This means that the first-recorded diagnosis of schizophrenia within the dataset may not necessarily represent the first episode of psychosis if patients had received care in other mental health services or within the same mental health service prior to digitization of healthcare records. Furthermore, while we were able to ascertain comorbid SUD diagnosis among patients with schizophrenia, we were unable to ascertain more detailed information on the chronicity and frequency of substance use as these data were not available within the dataset. While our analyses benefit from a large sample size reflecting RWE, the database is reliant on accurate input by mental health professionals in a clinical setting, however, EHR data are sometimes incomplete. For example, demographic variables such as gender, race, marital status, and employment status may not be recorded by clinicians, or may not be declared by patients, possibly introducing reporting bias. Sampling and recall bias could also be introduced given the location and type of clinical service, as well as the reliance on patient's recount of events to inform diagnoses (Camm and Fox, 2018).

Given the retrospective nature of the study, our results show associations only and are not indicative of causality. Although adjustments were made for confounding factors of age, gender, race, marital status, employment status, and year of diagnosis, there may have been other factors (including non-psychiatric comorbidities (Manuel et al., 2013) or differences in positive and negative symptoms) that could influence the clinical outcomes associated with schizophrenia and comorbid SUDs.

## Contributors

RP conceived the study. MV, GG, SK and GB conducted data analysis supervised by MR. RP, KC, EP, MV, GG, SK, GB, MER and SHK contributed to writing the manuscript. SHK had full access to the data and can take responsibility for the integrity of the data and the accuracy of the data analysis; the lead author (RP) affirms that the manuscript is an honest, accurate, and transparent account of the study being reported; that no important aspects of the study have been omitted; and that any discrepancies from the study as planned (and, if relevant, registered) have been explained.

## Role of funding source

The present study was sponsored by Holmusk Technologies, Inc.

## Declaration of competing interest

All authors report current or previous employment with Holmusk Technologies, Inc. RP has received grant funding from the National Institute of Health Research (NIHR301690), the Medical Research Council (MR/S003118/1), the Academy of Medical Sciences (SGL015/1020) and Janssen, and consulting fees from Holmusk, Akrivia Health, Columbia Data Analytics, Otsuka and Boehringer Ingelheim.

## Appendix A Supplementary data

Appendix A The RECORD statement – checklist of items, extended from the STROBE statement, that should be reported in observational studies using routinely collected health data. Appendix A

Supplementary material Image 1
