# Supplementary material for: Associations of comorbid substance use disorders with clinical outcomes in schizophrenia using electronic health record data
Source: Schizophr Res. 2023 Oct;260:191–7. doi: 10.1016/j.schres.2023.08.023 (PMC10881404; doi:10.1016/j.schres.2023.08.023)
Supplement: Supplementary file 2 — Supplementary material [file mmc2.docx]

**Associations of comorbid substance use disorders with clinical outcomes in schizophrenia using electronic health record data: Supplementary Materials**

**Supplementary Table 1:** Poisson distribution model comparing the impact of comorbid substance use disorders (SUD) on number of unique antipsychotics prescribed to patients with schizophrenia (n=13,634), a significance threshold of p<.05 was used.

| **Variables** |  |  |  |  |  |  | **Reference category** | |
| --- | --- | --- | --- | --- | --- | --- | --- | --- |
|  | n | Mean (SD) | IRR | Confidence Interval | *p* |  | n | Mean (SD) |
| SUD category |  |  |  |  |  |  | *Without SUD* | |
| Alcohol only | 540 | 1.3 (1.1) | 0.93 | 0.86 to 1.01 | 0.073 |  | 10624 | 1.4 (1.1) |
| Cannabis only | 363 | 1.6 (1.3) | 1.13 | 1.04 to 1.23 | 0.003 |  |  |  |
| Cocaine only | 135 | 1.1 (1.1) | 0.91 | 0.77 to 1.07 | 0.25 |  |  |  |
| Opioid only | 113 | 1.5 (1.1) | 1.04 | 0.89 to 1.21 | 0.62 |  |  |  |
| Nicotine only | 59 | 1.7 (1.2) | 1.20 | 0.98 to 1.46 | 0.076 |  |  |  |
| Stimulant only | 42 | 1.5 (1.0) | 0.95 | 0.74 to 1.22 | 0.68 |  |  |  |
| Others only | 667 | 1.4 (1.2) | 1.01 | 0.95 to 1.08 | 0.67 |  |  |  |
| Polysubstance | 1091 | 1.5 (1.3) | 1.08 | 1.03 to 1.14 | 0.003 |  |  |  |
|  |  |  |  |  |  |  |  |  |
| Gender |  |  |  |  |  |  | *Female* | |
| Male | 8571 | 1.3 (1.1) | 0.91 | 0.89 to 0.94 | < 0.001 |  | 5063 | 1.5 (1.2) |
|  |  |  |  |  |  |  |  |  |
| Age |  |  |  |  |  |  | *Age < 40* | |
| Age 40 and above | 7252 | 1.3 (1.1) | 0.89 | 0.86 to 0.92 | < 0.001 |  | 6382 | 1.5 (1.2) |
|  |  |  |  |  |  |  |  |  |
| Race |  |  |  |  |  |  | *Black* | |
| White | 5280 | 1.5 (1.3) | 1.14 | 1.1 to 1.18 | < 0.001 |  | 4259 | 1.4 (1.1) |
| Others | 1002 | 1.4 (1.2) | 1.04 | 0.98 to 1.1 | 0.18 |  |  |  |
| Unknown | 3093 | 1.2 (1.0) | 0.90 | 0.86 to 0.94 | < 0.001 |  |  |  |
|  |  |  |  |  |  |  |  |  |
| Marital status |  |  |  |  |  |  | *Divorced/*  *Separated* | |
| Single | 8694 | 1.4 (1.2) | 0.99 | 0.94 to 1.05 | 0.75 |  | 1152 | 1.4 (1.2) |
| Married/Engaged/In a relationship | 1066 | 1.5 (1.3) | 1.05 | 0.98 to 1.13 | 0.16 |  |  |  |
| Widowed | 237 | 1.4 (1.1) | 1.03 | 0.92 to 1.16 | 0.61 |  |  |  |
| Unknown | 2485 | 1.2 (1.0) | 0.92 | 0.86 to 0.98 | 0.008 |  |  |  |
|  |  |  |  |  |  |  |  |  |
| Employment status |  |  |  |  |  |  | *Disabled* | |
| Employed | 579 | 1.6 (1.4) | 0.95 | 0.87 to 1.03 | 0.18 |  | 1100 | 1.6 (1.2) |
| Unemployed/ Student/Retired | 2377 | 1.6 (1.2) | 0.93 | 0.87 to 0.98 | 0.008 |  |  |  |
| Unknown | 9578 | 1.3 (1.1) | 0.82 | 0.78 to 0.86 | < 0.001 |  |  |  |
|  |  |  |  |  |  |  |  |  |
| Year of Schizophrenia diagnosis |  |  |  |  |  |  | *2004 and before* | |
| 2005 - 2009 | 3375 | 1.4 (1.1) | 1.32 | 1.21 to 1.43 | < 0.001 |  | 586 | 1.0 (1.0) |
| 2010 – 2014 | 6941 | 1.4 (1.2) | 1.34 | 1.23 to 1.46 | < 0.001 |  |  |  |
| 2015 - 2020 | 2732 | 1.5 (1.2) | 1.45 | 1.33 to 1.58 | < 0.001 |  |  |  |

Note: IRR: Incidence rate ratio

**Supplementary Table 2:** CoxPH model comparing the impact of comorbid substance use disorders (SUD) on the duration of time to first antipsychotic discontinuation for patients with schizophrenia (n=9,504), a significance threshold of p<.05 was used.

| **Variables** |  |  |  |  |  |  | **Reference category** | |
| --- | --- | --- | --- | --- | --- | --- | --- | --- |
|  | n | Mean (SD) | Hazard Ratio | Confidence Interval | *p* |  | n | Mean (SD) |
| SUD category |  |  |  |  |  |  | *without SUD* | |
| Alcohol only | 346 | 342.1 (535.8) | 1.12 | 0.99 to 1.27 | 0.077 |  | 7490 | 357.8 (579.6) |
| Cannabis only | 267 | 306.9 (486.7) | 1.06 | 0.92 to 1.23 | 0.41 |  |  |  |
| Opioid only | 82 | 333.1 (543.4) | 1.21 | 0.95 to 1.56 | 0.13 |  |  |  |
| Cocaine only | 66 | 180.1 (356.4) | 1.87 | 1.42 to 2.45 | < 0.001 |  |  |  |
| Nicotine only | 48 | 488.0 (612.4) | 0.93 | 0.67 to 1.28 | 0.64 |  |  |  |
| Stimulant only | 31 | 167.0 (333.8) | 1.64 | 1.07 to 2.52 | 0.024 |  |  |  |
| Others only | 437 | 233.9 (440.5) | 1.39 | 1.24 to 1.56 | < 0.001 |  |  |  |
| Polysubstance | 737 | 219.4 (385.3) | 1.46 | 1.33 to 1.59 | < 0.001 |  |  |  |
|  |  |  |  |  |  |  |  |  |
| Gender |  |  |  |  |  |  | *Female* | |
| Male | 5829 | 367.4 (590.3) | 0.85 | 0.81 to 0.90 | < 0.001 |  | 5063 | 248.6 (502.4) |
|  |  |  |  |  |  |  |  |  |
| Age |  |  |  |  |  |  | *Age < 40* | |
| Age 40 and above | 5023 | 432.5 (640.8) | 0.74 | 0.71 to 0.78 | < 0.001 |  | 6382 | 192.4 (428.3) |
|  |  |  |  |  |  |  |  |  |
| Race |  |  |  |  |  |  | *Black* | |
| White | 4055 | 430.4 (632.7) | 0.86 | 0.82 to 0.91 | < 0.001 |  | 4259 | 210.5 (466.6) |
| Others | 701 | 218.6 (483.9) | 1.12 | 1.01 to 1.24 | 0.031 |  |  |  |
| Unknown | 1891 | 320.3 (528.0) | 0.92 | 0.86 to 0.99 | 0.025 |  |  |  |
|  |  |  |  |  |  |  |  |  |
| Marital status |  |  |  |  |  |  | *Divorced/*  *Separated* | |
| Single^a^ | - | - | - | - | - |  |  |  |
| Married/ Engaged/In a relationship | 780 | 405.4 (611.5) | 1.00 | 0.92 to 1.09 | 0.95 |  | 1152 | 347.8 (568.1) |
| Widowed | 180 | 352.2 (498.2) | 1.15 | 0.97 to 1.36 | 0.12 |  |  |  |
| Unknown^a^ | - | - | - | - | - |  |  |  |
|  |  |  |  |  |  |  |  |  |
| Employment status |  |  |  |  |  |  | *Disabled* | |
| Employed | 446 | 322.8 (602.2) | 0.90 | 0.79 to 1.03 | 0.12 |  | 1100 | 244.4 (457.5) |
| Unemployed/ Student/ Retired | 1823 | 198.3 (405.6) | 1.17 | 1.07 to 1.29 | < 0.001 |  |  |  |
| Unknown | 6367 | 412.5 (620.3) | 0.75 | 0.69 to 0.82 | < 0.001 |  |  |  |

^a^Dropped due to violation of proportional hazard assumption

**Supplementary Table 3:** Logistic regression for baseline CGI-S scores comparing patients with schizophrenia and a specific SUD versus the absence of a specific SUD (n=12676) CGI-S scores were dichotomized into two groups: low = 1-4 and high = 5-7, a significance threshold of p<.05 was used.

| **Variables** |  |  |  |  |  |  | **Reference category** | |
| --- | --- | --- | --- | --- | --- | --- | --- | --- |
|  | n | Mean (SD) | Odds Ratio | Confidence Interval | *p* |  | n | Mean (SD) |
| Specific SUD |  |  |  |  |  |  | *Absence of the specific SUD* | |
| Alcohol | 1092 | 4.5 (1.4) | 0.91 | 0.79 to 1.04 | 0.17 |  | 11584 | 4.6 (1.2) |
| Cannabis | 920 | 4.8 (1.1) | 1.30 | 1.11 to 1.51 | 0.001 |  | 11756 | 4.6 (1.2) |
| Cocaine | 421 | 4.7 (1.2) | 1.05 | 0.85 to 1.30 | 0.66 |  | 12255 | 4.6 (1.2) |
| Opioid | 271 | 4.4 (1.3) | 0.63 | 0.49 to 0.82 | < 0.001 |  | 12405 | 4.6 (1.2) |
| Nicotine | 241 | 4.5 (1.3) | 0.91 | 0.69 to 1.20 | 0.50 |  | 12435 | 4.6 (1.2) |
| Stimulant | 156 | 4.7 (1.1) | 1.35 | 0.95 to 1.90 | 0.091 |  | 12520 | 4.6 (1.2) |
| Others | 1099 | 4.9 (1.1) | 1.53 | 1.33 to 1.76 | < 0.001 |  | 11577 | 4.6 (1.2) |
|  |  |  |  |  |  |  |  |  |
| Gender |  |  |  |  |  |  | *Female* | |
| Male | 7939 | 4.6 (1.2) | 0.98 | 0.9 to 1.05 | 0.54 |  | 4737 | 4.6 (1.2) |
|  |  |  |  |  |  |  |  |  |
| Age |  |  |  |  |  |  | *Age < 40* | |
| Age 40 and above | 6725 | 4.5 (1.3) | 0.83 | 0.77 to 0.90 | < 0.001 |  | 5951 | 4.7 (1.2) |
|  |  |  |  |  |  |  |  |  |
| Race |  |  |  |  |  |  | *Black* | |
| White | 4890 | 4.6 (1.2) | 1.10 | 1.01 to 1.20 | 0.034 |  | 3848 | 4.6 (1.2) |
| Others | 988 | 5.1 (1.1) | 1.90 | 1.63 to 2.22 | < 0.001 |  |  |  |
| Unknown | 2950 | 4.4 (1.3) | 0.92 | 0.83 to 1.02 | 0.11 |  |  |  |
|  |  |  |  |  |  |  |  |  |
| Marital status |  |  |  |  |  |  | *Divorced/*  *Separated* | |
| Single | 8123 | 4.7 (1.2) | 1.41 | 1.23 to 1.61 | < 0.001 |  | 1068 | 4.4 (1.3) |
| Married/  Engaged/In a relationship | 981 | 4.4 (1.2) | 0.92 | 0.77 to 1.10 | 0.39 |  |  |  |
| Widowed | 208 | 4.4 (1.2) | 1.30 | 0.96 to 1.77 | 0.086 |  |  |  |
| Unknown | 2296 | 4.4 (1.3) | 1.10 | 0.94 to 1.28 | 0.23 |  |  |  |
|  |  |  |  |  |  |  |  |  |
| Employment status |  |  |  |  |  |  | *Disabled* | |
| Employed | 534 | 4.8 (1.2) | 0.90 | 0.72 to 1.13 | 0.37 |  | 1000 | 4.8 (1.1) |
| Unemployed/ Student/  Retired | 2207 | 4.9 (1.1) | 1.26 | 1.07 to 1.48 | 0.005 |  |  |  |
| Unknown | 8935 | 4.5 (1.3) | 0.67 | 0.59 to 0.77 | < 0.001 |  |  |  |
|  |  |  |  |  |  |  |  |  |
| Year of Schizophrenia diagnosis |  |  |  |  |  |  | *2004 and before* | |
| 2005 - 2009 | 3290 | 4.8 (1.2) | 0.99 | 0.74 to 1.32 | 0.94 |  | 216 | 4.8 (0.9) |
| 2010 – 2014 | 6560 | 4.5 (1.3) | 0.65 | 0.49 to 0.87 | 0.003 |  |  |  |
| 2015 - 2020 | 2610 | 4.5 (1.2) | 0.57 | 0.42 to 0.76 | < 0.001 |  |  |  |

**Supplementary Table 4:** Poisson distribution model for number of inpatient days comparing patients with schizophrenia and a specific SUD versus the absence of a specific SUD (n=13,634) a significance threshold of p<.05 was used.

| **Variables** |  |  |  |  |  |  | **Reference category** | |
| --- | --- | --- | --- | --- | --- | --- | --- | --- |
|  | n | Mean (SD) | IRR | Confidence Interval | *p* |  | n | Mean (SD) |
| Specific SUD |  |  |  |  |  |  | *Absence of the specific SUD* | |
| Alcohol | 1204 | 5.9 (13.1) | 0.85 | 0.83 to 0.87 | < 0.001 |  | 12430 | 7.8 (20.0) |
| Cannabis | 988 | 8.0 (16.7) | 1.04 | 1.01 to 1.06 | 0.003 |  | 12646 | 7.6 (19.7) |
| Cocaine | 506 | 5.4 (10.9) | 0.77 | 0.74 to 0.8 | < 0.001 |  | 13128 | 7.7 (19.8) |
| Opioid | 280 | 5.4 (12.8) | 0.76 | 0.72 to 0.8 | < 0.001 |  | 13354 | 7.6 (19.7) |
| Nicotine | 252 | 4.7 (11.5) | 0.71 | 0.67 to 0.75 | < 0.001 |  | 13382 | 7.6 (19.7) |
| Stimulant | 158 | 7.6 (13.4) | 1.07 | 1.01 to 1.14 | 0.017 |  | 13476 | 7.6 (19.6) |
| Others | 1129 | 10.3 (18.6) | 1.37 | 1.35 to 1.4 | < 0.001 |  | 12505 | 7.3 (19.6) |
|  |  |  |  |  |  |  |  |  |
| Gender |  |  |  |  |  |  | *Female* | |
| Male | 8571 | 7.6 (19.8) | 0.93 | 0.92 to 0.94 | < 0.001 |  | 5063 | 7.5 (19.1) |
|  |  |  |  |  |  |  |  |  |
| Age |  |  |  |  |  |  | *Age < 40* | |
| Age 40 and above | 7525 | 7.2 (20.1) | 1.16 | 1.14 to 1.17 | < 0.001 |  | 6382 | 8.0 (18.9) |
|  |  |  |  |  |  |  |  |  |
| Race |  |  |  |  |  |  | *Black* | |
| White | 5280 | 7.2 (19.3) | 1.0 | 0.98 to 1.01 | 0.742 |  | 4259 | 7.8 (18.9) |
| Others | 1002 | 16.4 (29.7) | 1.9 | 1.86 to 1.93 | < 0.001 |  |  |  |
| Unknown | 3093 | 5.1 (15.4) | 0.84 | 0.82 to 0.85 | < 0.001 |  |  |  |
|  |  |  |  |  |  |  |  |  |
| Marital status |  |  |  |  |  |  | *Divorced/*  *Separated* | |
| Single | 8694 | 9.8 (22.5) | 2.44 | 2.36 to 2.52 | < 0.001 |  | 1152 | 3.6 (9.4) |
| Married/Engaged/In a relationship | 1066 | 3.3 (9.6) | 0.9 | 0.86 to 0.94 | < 0.001 |  |  |  |
| Widowed | 237 | 4.6 (10.4) | 1.25 | 1.17 to 1.34 | < 0.001 |  |  |  |
| Unknown | 2485 | 3.9 (13.9) | 1.27 | 1.22 to 1.32 | < 0.001 |  |  |  |
|  |  |  |  |  |  |  |  |  |
| Employment status |  |  |  |  |  |  | *Disabled* | |
| Employed | 579 | 6.5 (12.5) | 0.65 | 0.63 to 0.68 | < 0.001 |  | 1100 | 9.6 (21.5) |
| Unemployed/ Student/Retired | 2377 | 13.8 (26.7) | 1.32 | 1.29 to 1.35 | < 0.001 |  |  |  |
| Unknown | 9578 | 5.9 (17.0) | 0.66 | 0.65 to 0.68 | < 0.001 |  |  |  |
|  |  |  |  |  |  |  |  |  |
| Year of Schizophrenia diagnosis |  |  |  |  |  |  | *2004 and before* | |
| 2005 - 2009 | 3375 | 8.3 (21.5) | 1.57 | 1.51 to 1.64 | < 0.001 |  | 586 | 4.1 (8.3) |
| 2010 – 2014 | 6941 | 7.3 (19.8) | 1.47 | 1.41 to 1.53 | < 0.001 |  |  |  |
| 2015 - 2020 | 2732 | 8.3 (17.8) | 1.54 | 1.47 to 1.6 | < 0.001 |  |  |  |

Note: IRR: Incidence rate ratio

**Supplementary Table 5:** Poisson distribution model for number of unique antipsychotics prescribed comparing patients with schizophrenia and a specific SUD versus the absence of a specific SUD (n=13634), a significance threshold of p<.05 was used.

| **Variables** |  |  |  |  |  |  | **Reference category** | |
| --- | --- | --- | --- | --- | --- | --- | --- | --- |
|  | n | Mean (SD) | IRR | Confidence Interval | *p* |  | n | Mean (SD) |
| Specific SUD |  |  |  |  |  |  | *Absence of the specific SUD* | |
| Alcohol | 1204 | 1.4 (1.2) | 0.97 | 0.92 to 1.03 | 0.30 |  | 12430 | 1.4 (1.1) |
| Cannabis | 988 | 1.5 (1.3) | 1.09 | 1.02 to 1.15 | 0.005 |  | 12646 | 1.4 (1.1) |
| Cocaine | 506 | 1.3 (1.2) | 0.93 | 0.85 to 1.01 | 0.075 |  | 13128 | 1.4 (1.2) |
| Opioid | 280 | 1.5 (1.3) | 1.00 | 0.91 to 1.11 | 0.96 |  | 13354 | 1.4 (1.2) |
| Nicotine | 252 | 1.7 (1.2) | 1.16 | 1.05 to 1.29 | 0.003 |  | 13382 | 1.4 (1.2) |
| Stimulant | 158 | 1.7 (1.6) | 1.04 | 0.92 to 1.18 | 0.50 |  | 13476 | 1.4 (1.2) |
| Others | 1129 | 1.5 (1.3) | 1.07 | 1.02 to 1.13 | 0.009 |  | 12505 | 1.4 (1.1) |
|  |  |  |  |  |  |  |  |  |
| Gender |  |  |  |  |  |  | *Female* | |
| Male | 8571 | 1.4 (1.1) | 0.91 | 0.88 to 0.94 | < 0.001 |  | 5063 | 1.5 (1.2) |
|  |  |  |  |  |  |  |  |  |
| Age |  |  |  |  |  |  | *Age < 40* | |
| Age 40 and above | 7252 | 1.3 (1.1) | 0.89 | 0.87 to 0.92 | < 0.001 |  | 6382 | 1.6 (1.2) |
|  |  |  |  |  |  |  |  |  |
| Race |  |  |  |  |  |  | *Black* | |
| White | 5280 | 1.5 (1.3) | 1.14 | 1.10 to 1.18 | < 0.001 |  | 4259 | 1.4 (1.1) |
| Others | 1002 | 1.4 (1.2) | 1.04 | 0.98 to 1.10 | 0.22 |  |  |  |
| Unknown | 3093 | 1.2 (1.0) | 0.90 | 0.86 to 0.94 | < 0.001 |  |  |  |
|  |  |  |  |  |  |  |  |  |
| Marital status |  |  |  |  |  |  | *Divorced/*  *Separated* | |
| Single | 8694 | 1.4 (1.2) | 0.99 | 0.94 to 1.05 | 0.78 |  | 1152 | 1.4 (1.2) |
| Married/Engaged/In a relationship | 1066 | 1.5 (1.3) | 1.05 | 0.98 to 1.13 | 0.14 |  |  |  |
| Widowed | 237 | 1.4 (1.1) | 1.03 | 0.92 to 1.16 | 0.62 |  |  |  |
| Unknown | 2485 | 1.2 (1.0) | 0.92 | 0.86 to 0.98 | 0.009 |  |  |  |
|  |  |  |  |  |  |  |  |  |
| Employment status |  |  |  |  |  |  | *Disabled* | |
| Employed | 579 | 1.6 (1.4) | 0.94 | 0.87 to 1.02 | 0.16 |  | 1100 | 1.6 (1.2) |
| Unemployed/ Student/Retired | 2377 | 1.6 (1.2) | 0.92 | 0.87 to 0.98 | 0.006 |  |  |  |
| Unknown | 9578 | 1.3 (1.1) | 0.82 | 0.78 to 0.86 | < 0.001 |  |  |  |
|  |  |  |  |  |  |  |  |  |
| Year of Schizophrenia diagnosis |  |  |  |  |  |  | *2004 and before* | |
| 2005 - 2009 | 3375 | 1.35 (1.07) | 1.30 | 1.19 to 1.42 | < 0.001 |  | 586 | 1.0 (1.0) |
| 2010 – 2014 | 6941 | 1.40 (1.17) | 1.32 | 1.22 to 1.44 | < 0.001 |  |  |  |
| 2015 - 2020 | 2732 | 1.52 (1.22) | 1.43 | 1.31 to 1.56 | < 0.001 |  |  |  |

Note: IRR: Incidence rate ratio

**Supplementary Table 6:** CoxPH model for the duration of time to first antipsychotic discontinuation comparing patients with schizophrenia and a specific SUD versus the absence of a specific SUD (n=9,504), a significance threshold of p<.05 was used.

| **Variables** |  |  |  |  |  |  | **Reference category** | |
| --- | --- | --- | --- | --- | --- | --- | --- | --- |
|  | n (days) | Mean (SD) | Hazard Ratio | Confidence Interval | *p* |  | n (days) | Mean (SD) |
| Specific SUD |  |  |  |  |  |  | *Absence of the specific SUD* | |
| Alcohol | 800 | 295.4 (482.7) | 1.06 | 0.96 to 1.16 | 0.24 |  | 12430 | 254.6 (519.0) |
| Cannabis | 707 | 242.3 (414.5) | 1.09 | 0.99 to 1.21 | 0.072 |  | 12646 | 255.4 (520.2) |
| Cocaine | 281 | 181.6 (360.0) | 1.35 | 1.17 to 1.57 | < 0.001 |  | 13128 | 255.1 (517.0) |
| Opioid | 190 | 251.5 (433.7) | 1.16 | 0.98 to 1.38 | 0.078 |  | 13354 | 251.1 (513.4) |
| Nicotine^a^ | - | - | - | - | - |  | - | - |
| Stimulant | 117 | 174.2 (335.6) | 1.15 | 0.92 to 1.44 | 0.23 |  | 13476 | 250.8 (512.9) |
| Others | 768 | 192.2 (368.4) | 1.42 | 1.30 to 1.56 | < 0.001 |  | 12505 | 260.1 (523.8) |
|  |  |  |  |  |  |  |  |  |
| Gender |  |  |  |  |  |  | *Female* | |
| Male | 5829 | 367.4 (590.3) | 0.85 | 0.81 to 0.90 | < 0.001 |  | 5063 | 248.6 (502.4) |
|  |  |  |  |  |  |  |  |  |
| Age |  |  |  |  |  |  | *Age < 40* | |
| Age 40 and above | 5023 | 432.5 (640.8) | 0.74 | 0.71 to 0.78 | < 0.001 |  | 6382 | 192.4 (428.3) |
|  |  |  |  |  |  |  |  |  |
| Race |  |  |  |  |  |  | *Black* | |
| White | 4055 | 430.4 (632.7) | 0.87 | 0.82 to 0.92 | < 0.001 |  | 4259 | 210.5 (466.6) |
| Others | 701 | 218.6 (483.9) | 1.13 | 1.02 to 1.25 | 0.025 |  |  |  |
| Unknown | 1891 | 320.3 (528.0) | 0.92 | 0.86 to 0.99 | 0.030 |  |  |  |
|  |  |  |  |  |  |  |  |  |
| Marital status |  |  |  |  |  |  | *Divorced/Separated* | |
| Single^a^ | - | - | - | - | - |  | 1152 | 347.8 (568.1) |
| Married/ Engaged/In a relationship | 780 | 405.4 (611.5) | 0.99 | 0.91 to 1.08 | 0.89 |  |  |  |
| Widowed | 180 | 352.2 (498.2) | 1.15 | 0.97 to 1.36 | 0.12 |  |  |  |
| Unknown^a^ | - | - | - | - | - |  |  |  |
|  |  |  |  |  |  |  |  |  |
| Employment status |  |  |  |  |  |  | *Disabled* | |
| Employed | 446 | 322.8 (602.2) | 0.89 | 0.78 to 1.02 | 0.086 |  | 1100 | 244.4 (457.5) |
| Unemployed/ Student/ Retired | 1823 | 198.3 (405.6) | 1.17 | 1.06 to 1.28 | 0.001 |  |  |  |
| Unknown | 6367 | 412.5 (620.3) | 0.75 | 0.69 to 0.82 | < 0.001 |  |  |  |

^a^Dropped due to violation of proportional hazard assumption

**Supplementary Table 7:** ICD9/10 codes used to identify each psychiatric disorder

| **Disorder** | **ICD-9/10 Code** |
| --- | --- |
| Schizophrenia | F20, F20.8, F20.0, F20.1, F20.2, F20.3, F20.5, F20.9, 295.94, 295.00, 295.15, 295.53, 295.6, 295.30, 295.31, 295.54, 295.64, 295.95, 295.02, 295.25, 295.34, 295.10, 295.4, 295.42, 295.81, 295.9, 295.12, 295.91, 295.92, 295.01, 295.11, 295.14, 295.20, 295.33, 295.35, 295.8, 295.22, 295.32, 295.40, 295.52, 295.55, 295.63, 295.03, 295.23, 295.44, 295.51, 295.65, 295.5, 295.62, 295.90, 295.1, 295.83, 295.0, 295.04, 295.13, 295.24, 295.61, 295.84, 295.82, 295.21, 295.93, 295.05, 295.2, 295.3, 295.41, 295.43, 295.45, 295.50, 295.85, F20.89, F20.81 |
| Alcohol use disorder | F10.10, F10.11, F10.120, F10.121, F10.129, F10.14, F10.15, F10.150, F10.151, F10.159, F10.18, F10.180, F10.181, F10.182, F10.188, F10.19, F10.2, F10.20, F10.21, F10.22, F10.220, F10.221, F10.229, F10.23, F10.230, F10.231, F10.232, F10.239, F10.24, F10.25, F10.250, F10.251, F10.259, F10.26, F10.27, F10.28, F10.280, F10.281, F10.282, F10.288, F10.29, F10.92, F10.920, F10.921, F10.929, F10.94, F10.95, F10.950, F10.951, F10.959, F10.96, F10.97, F10.98, F10.980, F10.981, F10.982, F10.988, F10.99  291, 291, 291.1, 291.2, 291.3, 291.4, 291.5, 291.8, 291.81, 291.82, 291.89, 291.9, 303, 303, 303, 303.01, 303.02, 303.03, 303.9, 303.9.0, 303.91, 303.92, 303.93, 305, 305, 305.01, 305.02, 305.03 |
| Cannabis use disorder | F12, F12.10, F12.11, F12.12, F12.120, F12.121, F12.122, F12.129, F12.15, F12.150, F12.151, F12.159, F12.18, F12.180, F12.188, F12.19, F12.20, F12.21, F12.22, F12.220, F12.221, F12.222, F12.229, F12.25, F12.250, F12.251, F12.259, F12.28, F12.280, F12.288, F12.29, F12.90, F12.92, F12.920, F12.921, F12.922, F12.929, F12.95, F12.950, F12.951, F12.959, F12.98, F12.980, F12.988, F12.99  304.3, 304.3, 304.31, 304.32, 304.33, 305.2, 305.2, 305.21, 305.22, 305.23 |
| Cocaine use disorder | F14.10, F14.11, F14.12, F14.120, F14.121, F14.122, F14.129, F14.14, F14.15, F14.150, F14.151, F14.159, F14.18, F14.180, F14.181, F14.182, F14.188, F14.19, F14.2, F14.20, F14.21, F14.22, F14.220, F14.221, F14.222, F14.229, F14.23, F14.24, F14.25, F14.250, F14.251, F14.259, F14.28, F14.280, F14.281, F14.282, F14.288, F14.29, F14.90, F14.92, F14.920, F14.921, F14.922, F14.929, F14.94, F14.95, F14.950  304.2, 304.2, 304.21, 304.22, 304.23, 305.6, 305.6, 305.61, 305.62, 305.63 |
| Opioid use disorder | F11.10, F11.11, F11.12, F11.120, F11.121, F11.122, F11.129, F11.14, F11.150, F11.151, F11.159, F11.18, F11.181, F11.182, F11.188, F11.19, F11.2, F11.20, F11.21, F11.220, F11.221, F11.222, F11.229, F11.23, F11.24, F11.25, F11.250, F11.251, F11.259, F11.28, F11.281, F11.282, F11.288, F11.29, F11.9, F11.90, F11.92, F11.920, F11.921, F11.922, F11.929, F11.93  304, 304, 304.01, 304.02, 304.03, 304.7, 304.7, 304.71, 304.72, 304.73, 305.5, 305.5, 305.51, 305.52, 305.53, |
| Nicotine use disorder | F17, F17.2, F17.20, F17.200, F17.201, F17.203, F17.208, F17.209, F17.21, F17.210, F17.211, F17.213, F17.218, F17.219, F17.22, F17.220, F17.221, F17.223, F17.228, F17.229, F17.29, F17.290, F17.291, F17.293, F17.298, F17.299  305.1 |
| Stimulant use disorder | F15.10, F15.11, F15.12, F15.120, F15.121, F15.122, F15.129, F15.14, F15.15, F15.150, F15.151, F15.159, F15.18, F15.180, F15.181, F15.182, F15.188, F15.19, F15.2, F15.20, F15.21, F15.22, F15.220, F15.221, F15.222, F15.229, F15.23, F15.24, F15.25, F15.250, F15.251, F15.259, F15.28, F15.280, F15.281, F15.282, F15.288, F15.29, F15.9, F15.90, F15.92, F15.920, F15.921, F15.922, F15.929, F15.93, F15.94, F15.95, F15.950, F15.951, F15.959, F15.98, F15.980, F15.981, F15.982, F15.988, F15.99  304.4, 304.4, 304.41, 304.42, 304.43, 305.7, 305.7, 305.71, 305.72, 305.73 |
| Sedatives/hypnotics use disorder | F13.10, F13.11, F13.12, F13.120, F13.121, F13.129, F13.14, F13.15, F13.150, F13.151, F13.159, F13.18, F13.180, F13.181, F13.182, F13.188, F13.19, F13.2, F13.20, F13.21, F13.22, F13.220, F13.221, F13.229, F13.23, F13.230, F13.231, F13.232, F13.239, F13.24, F13.25, F13.250, F13.251, F13.259, F13.26, F13.27, F13.28, F13.280, F13.281, F13.282, F13.288, F13.29, F13.9, F13.90, F13.92, F13.920, F13.921  304.1, 304.1, 304.11, 304.12, 304.13, 305.4, 305.4, 305.41, 305.42, 305.43 |
| Inhalant use disorder | F18, F18.1, F18.10, F18.11, F18.12, F18.120, F18.121, F18.129, F18.14, F18.15, F18.150, F18.151, F18.159, F18.17, F18.18, F18.180, F18.188, F18.19, F18.2, F18.20, F18.21, F18.22, F18.220, F18.221, F18.229, F18.24, F18.25, F18.250, F18.251, F18.259, F18.27, F18.28, F18.280, F18.288, F18.29, F18.9, F18.90, F18.92, F18.920, F18.921, F18.929, F18.94, F18.95, F18.950, F18.951, F18.959, F18.97, F18.98, F18.980, F18.988, F18.99 |
| Hallucinogen used disorder | F16.10, F16.11, F16.12, F16.120, F16.121, F16.122, F16.129, F16.14, F16.15, F16.150, F16.151, F16.159, F16.18, F16.180, F16.183, F16.188, F16.19, F16.2, F16.20, F16.21, F16.22, F16.220, F16.221, F16.229, F16.24, F16.25, F16.250, F16.251, F16.259, F16.28, F16.280, F16.283, F16.288, F16.29, F16.9, F16.90, F16.920, F16.921, F16.929, F16.94, F16.95, F16.950, F16.951, F16.959, F16.98, F16.980, F16.983, F16.988, F16.99  304.5, 304.5, 304.51, 304.52, 304.53, 305.3, 305.3, 305.31, 305.32, 305.33 |
| Others use disorders | F19.10, F19.11, F19.12, F19.120, F19.121, F19.122, F19.129, F19.14, F19.15, F19.150, F19.151, F19.159, F19.16, F19.17, F19.18, F19.180, F19.181, F19.182, F19.188  292, 292, 292.1, 292.11, 292.12, 292.2, 292.8, 292.81, 292.82, 292.83, 292.84, 292.85, 292.89, 292.9, 304.6, 304.6, 304.61, 304.62, 304.63, 304.8, 304.8, 304.81, 304.82, 304.83, 304.9, 304.9, 304.91, 304.92, 304.93, 305.8, 305.8, 305.81, 305.82, 305.83, 305.9, 305.9, 305.91, 305.92, 305.93 |

**Supplementary Figure 1:** Schematic of patient inclusion and exclusion criteria from the data available in the NeuroBlu database.

**
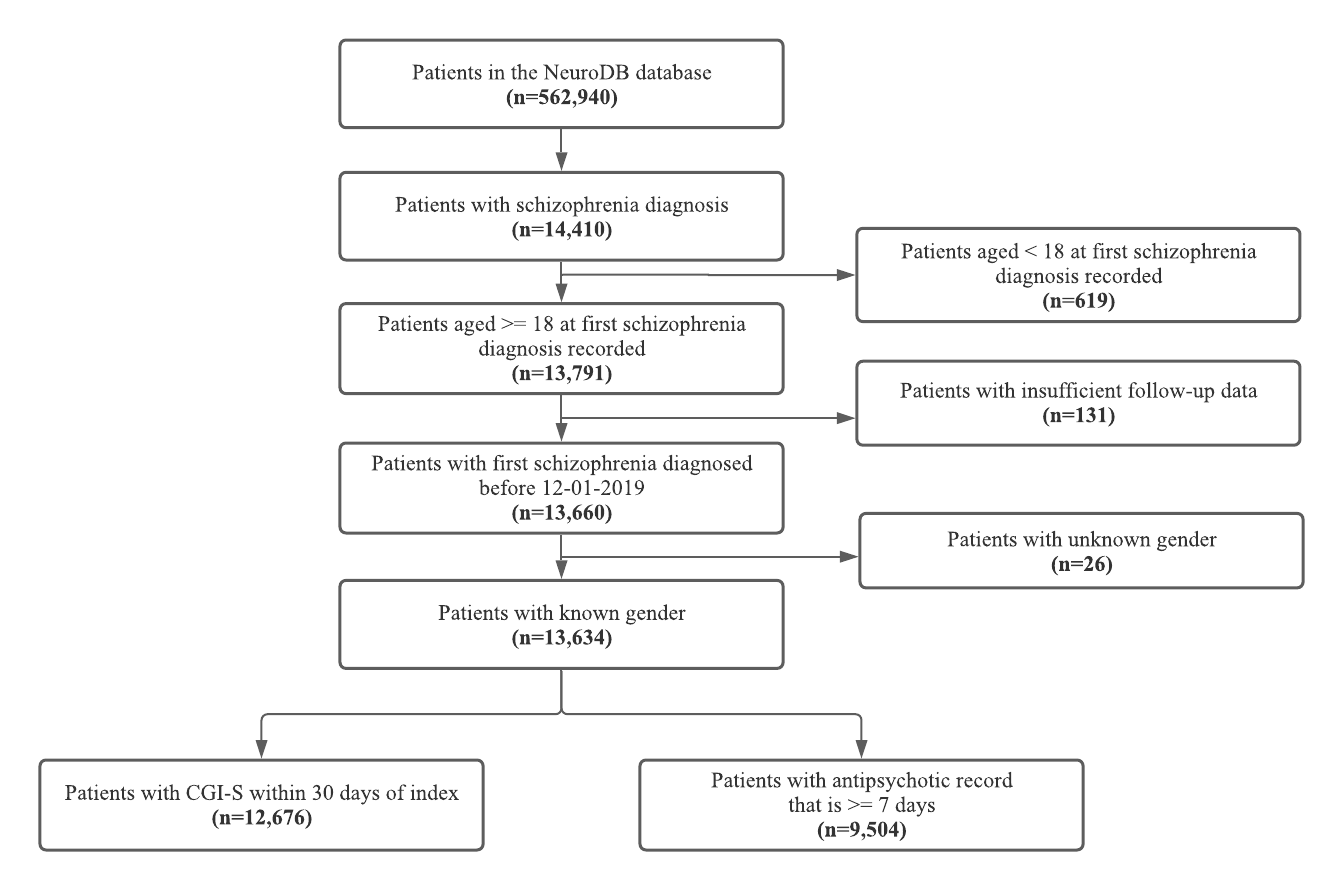
**

**Supplementary Figure 2:** Number of patients with schizophrenia with record of 1 or more substance use disorder diagnoses. This data was extracted from the electronic health records in the NeuroBlu database of n=3,010 patients before or at the time of schizophrenia diagnosis.


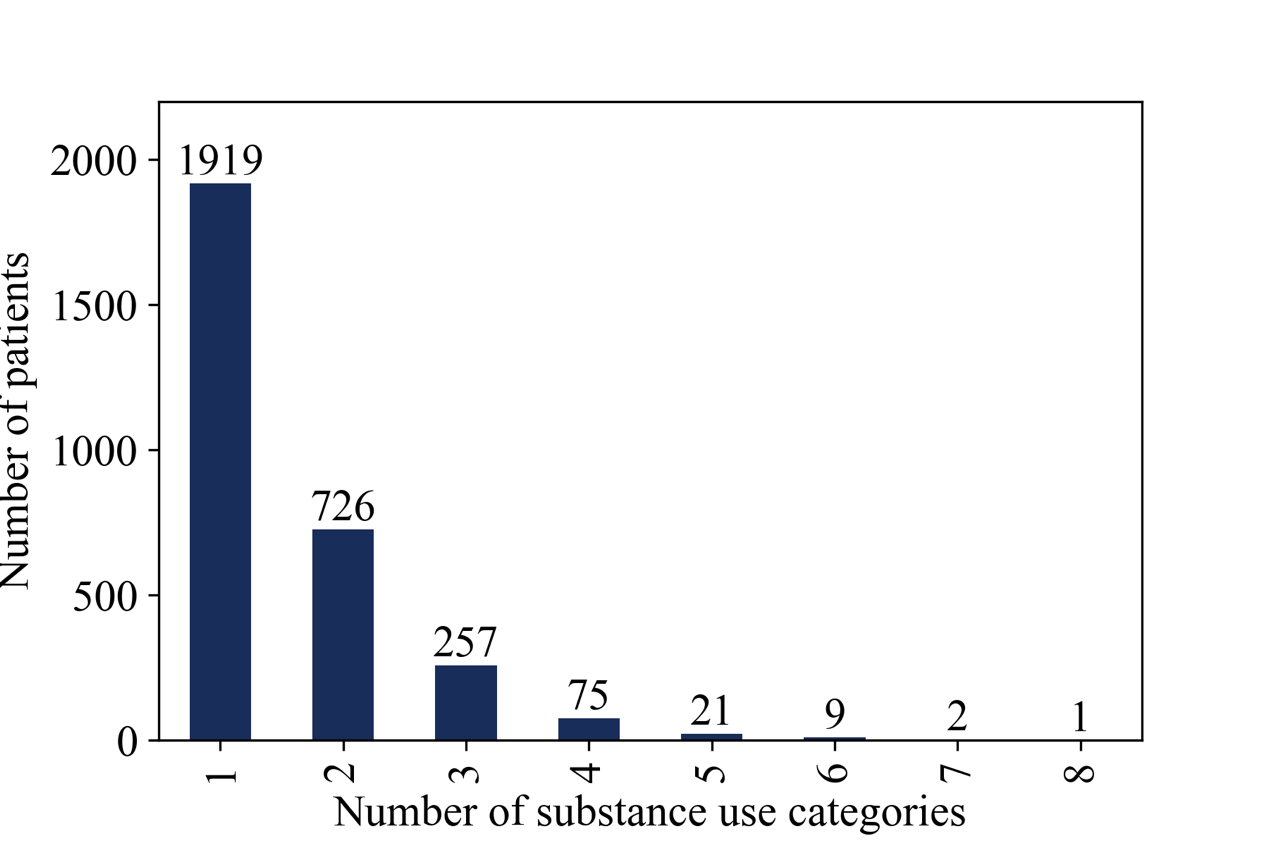


**Supplementary Figure 3:** Age was modelled in a generalized additive model to model age non-linearly in association to the odds of having a more severe baseline CGI-S (CGI-S of 5-7)


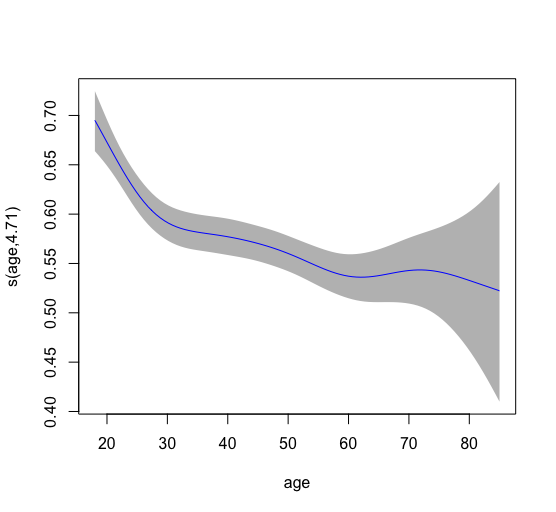


When modelled linearly, patients aged 40 and above were less likely to have a severe baseline CGI-S (OR=0.79, 95%CI [0.73, 0.86], *p*< .001). When modelled non-linearly, the overall findings remained consistent. Additionally, more nuances are revealed with a steep increase in the probability of having a severe baseline CGI-S from age 30 to 20.**Supplementary Figure 4:** Age was modelled in a generalized additive model to model age non-linearly in association to the number of inpatient days


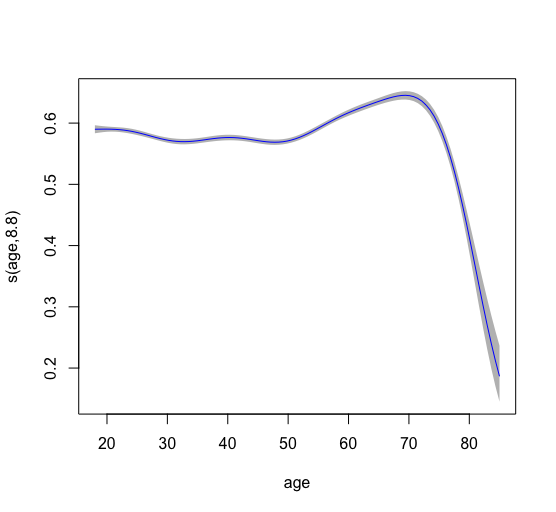


When modelled linearly, age was not found to be associated with number of inpatient days (IRR=1.01, 95%CI [1.00, 1.03], *p*=0.074). When modelled non-linearly, it appears that patients aged 40 to 70 were associated with greater number of inpatient days, and patients aged above 70 were associated with lesser number of inpatient days; though, it could be affected by the sample size as observed by the increase in confidence band.
